# A highway to carcinogenesis: the role of IQGAP1, a signaling scaffolding protein, in head and neck cancer development

**DOI:** 10.18632/oncoscience.511

**Published:** 2020-06-07

**Authors:** Tao Wei, Paul F. Lambert

**Affiliations:** ^1^McArdle Laboratory for Cancer Research, University of Wisconsin School of Medicine and Public Health, Madison, WI, USA

**Keywords:** head and neck cancer, IQGAP1, PI3K signaling, mouse model, scaffolding protein

## Abstract

Head and neck squamous cell carcinoma (HNSCC) is the sixth most frequent cancer worldwide. One of the most critical signaling pathways in HNSCC is the Epidermal Growth Factor Receptor/ Phosphatidylinositol 3-Kinase (EGFR/PI3K) pathway. IQ motif-containing GTPase- activating protein 1 (IQGAP1), a protein upregulated in multiple types of cancer, acts as a scaffold for this pathway and others implicated in cancer. IQGAP1 is overexpressed in HNSCCs, and its overexpression correlates with poorer prognosis in HNSCC patients, indicating that IQGAP1 might be important in HNSCC development. Here, we summarized our recent demonstrating a role of IQGAP1 in promoting HNSCC, at least in part, by scaffolding the EGFR/PI3K signaling pathway.

HNSCCs, which arise in the mouth and throat region, are the sixth most frequent cancer worldwide, with approximately 53,000 new cases and 11,000 associated deaths in the United States in 2019 [1]. The 5-year survival rate for head and neck cancer patients is about 60% [1]. Activation of the EGFR/PI3K pathway is observed in up to 74% of HNSCCs [2, 3]. *PIK3CA*, which encodes for the catalytic subunit of PI3K, is amplified in > 40% of HNSCCs, and contains gain-of function mutations in about 20% of HNSCCs [2, 4, 5]. These *PIK3CA* mutations correlate with advanced-stage HNSCCs, promoting HNSCC cell growth, tumor progression, invasion and metastasis [6-10]. Unfortunately, the efficacy of targeted therapies involving small molecule inhibitors of the EGFR/PI3K pathway has been limited due to the toxicity and possible drug resistance, raising the urgency of searching for other drug targets in the EGFR/PI3K signaling for more effective treatment of HNSCC patients [11, 12].

IQ motif-containing GTPase-activating protein 1, (IQGAP1), is a scaffolding protein that speeds up the efficiency of intracellular signaling by assembling multiple factors that mediate these signaling pathways. IQGAP1 affects multiple cellular activities such as cytoskeletal dynamics, cell-cell adhesion, cell proliferation, cell motility and invasion [13-16]. IQGAP1 is overexpressed in many human cancers, including breast, lung, colorectal cancers and HNSCCs [13, 16-18]. In HNSCCs, high levels of IQGAP1 expression correlates with poorer prognosis for the patients [18, 19]. IQGAP1 binds directly to EGFR and facilitates its ligand-dependent activation [20]. It also acts as a scaffold for the PI3K signaling pathway that is downstream of EGFR by assembling all of the kinases mediating production of phosphatidylinositol (3, 4, 5)-trisphophate (PIP3) upon EGFR receptor activation, which in turn results in increased phosphorylation of AKT (the activated form of AKT), a downstream effector of EGFR/PI3K signaling [21]. In a previous study, IQ3 peptide, a cell permeable peptide containing the PI3K binding motif on IQGAP1, was designed to specifically block the interaction between IQGAP1 and PI3K, and therefore inhibits IQGAP1-mediated PI3K signaling [21]. Treatment of HNSCC cell lines with IQ3 peptide, suppressed PI3K signaling, and inhibited cell survival, proliferation, migration and invasion, indicating that IQGAP1-mediated PI3K signaling is critical for human HNSCC cells [19, 22]. Reducing levels of IQGAP1 also resulted in decreased levels of phosphorylated-AKT (pAKT) in human HNSCC cell lines [19]. Likewise we found that mice germ-line deficient for IQGAP1 (*Iqgap1^-/-^*, [23]) showed reduced levels of both pAKT and phosphorylated-S6 (pS6, downstream of AKT), compared to wild type (*Iqgap1^+/+^*) mice, when stimulated with EGF [19], demonstrating that IQGAP1 contributes to the efficiency of the EGFR/PI3K signaling pathway *in vivo*.

Considering the importance of PI3K signaling in HNSCCs, we explored whether IQGAP1 plays a role in head and neck carcinogenesis using a well-validated mouse model that drives HNSCC using a synthetic oral carcinogen, 4-nitroquinoline 1-oxide (4NQO, [24]). After 4NQO treatment, *Iqgap1^-/-^* mice developed significantly lower cancer incidences, lesser disease severity, and fewer cancer foci per mouse, when compared to the *Iqgap1^+/+^* mice [19]. Tumors arising in *Iqgap1^-/-^* mice showed significantly lower levels of PI3K signaling than those in *Iqgap1^+/+^* mice, indicating that IQGAP1 contributes to HNSCC, at least in part, through PI3K signaling [19]. In human HNSCCs samples, levels of PI3K signaling correlates positively with levels of IQGAP1, further supporting the link between IQGAP1 and PI3K signaling in HNSCCs [19].

Other than increasing PI3K signaling, there are other possible mechanisms by which IQGAP1 may drive HNSCCs. In skin, IQGAP1 promotes tumorigenesis by scaffolding the Ras-MAPK signaling pathway [25]. However, in our study, we observed a reduction of the Ras-MAPK signaling in tumors compared to adjacent normal areas, regardless of IQGAP1 status [19] indicating that activation of this pathway might not be critical in HNSCC at least in this mouse model. IQGAP1 also mediates Wnt signaling by binding to multiple components along the Wnt pathway that mediate Wnt-dependent transcription [13, 26]. In a subset of HNSCC, increased β-catenin, a downstream effector and transcription factor for Wnt signaling, was observed in cancer cells compared to normal tissue [27]. This leaves open the possibility that IQGAP1 could also be contributing to HNSCC by mediating Wnt signaling pathway.

IQGAP1 binds to both wild type and mutated PI3K [21]. Both breast cancer and HNSCC cell lines carrying *PIK3CA* mutations or wild-type *PIK3CA* respond to IQ3 peptide treatment [19, 21]. This makes the IQGAP1-PI3K interaction a potential drug target for HNSCC patients with either wild type or mutant PI3K. *PIK3CA* mutation also correlates with shorter time to disease recurrence in a subset of HNSCC [28]. These HNSCC patients could potentially benefit from drugs targeting the IQGAP1-PI3K interaction, such as the IQ3 peptide or a peptidomimetic small molecule. More studies are needed to understand the underlying mechanism(s) by how IQGAP1 contributes to HNSCC, which will shed more lights on hunting for new HNSCC therapeutic targets.

## References

[1] American Cancer Society (2019). Cancer Facts and Figures 2019.

[2] Morris LG, Taylor BS, Bivona TG, Gong Y, Eng S, Brennan CW, Kaufman A, Kastenhuber ER, Banuchi VE, Singh B, Heguy A, Viale A, Mellinghoff IK (2011). Genomic dissection of the epidermal growth factor receptor (EGFR)/PI3K pathway reveals frequent deletion of the EGFR phosphatase PTPRS in head and neck cancers.. Proc Natl Acad Sci USA.

[3] Iglesias-Bartolome R, Martin D, Gutkind JS (2013). Exploiting the head and neck cancer oncogenome: widespread PI3K-mTOR pathway alterations and novel molecular targets.. Cancer Discov.

[4] Lui VW, Hedberg ML, Li H, Vangara BS, Pendleton K, Zeng Y, Lu Y, Zhang Q, Du Y, Gilbert BR, Freilino M, Sauerwein S, Peyser ND (2013). Frequent mutation of the PI3K pathway in head and neck cancer defines predictive biomarkers.. Cancer Discov.

[5] Cancer Genome Atlas Network (2015). Comprehensive genomic characterization of head and neck squamous cell carcinomas.. Nature.

[6] Estilo CL, O-Charoenrat P, Ngai I, Patel SG, Reddy PG, Dao S, Shaha AR, Kraus DH, Boyle JO, Wong RJ, Pfister DG, Huryn JM, Zlotolow IM (2003). The role of novel oncogenes squamous cell carcinoma-related oncogene and phosphatidylinositol 3-kinase p110α in squamous cell carcinoma of the oral tongue.. Clin Cancer Res.

[7] Fenic I, Steger K, Gruber C, Arens C, Woenckhaus J (2007). Analysis of PIK3CA and Akt/protein kinase B in head and neck squamous cell carcinoma.. Oncol Rep.

[8] Kozaki K, Imoto I, Pimkhaokham A, Hasegawa S, Tsuda H, Omura K, Inazawa J (2006). PIK3CA mutation is an oncogenic aberration at advanced stages of oral squamous cell carcinoma.. Cancer Sci.

[9] Woenckhaus J, Steger K, Werner E, Fenic I, Gamerdinger U, Dreyer T, Stahl U (2002). Genomic gain of PIK3CA and increased expression of p110alpha are associated with progression of dysplasia into invasive squamous cell carcinoma.. J Pathol.

[10] Du L, Chen X, Cao Y, Lu L, Zhang F, Bornstein S, Li Y, Owens P, Malkoski S, Said S, Jin F, Kulesz-Martin M, Gross N (2016). Overexpression of PIK3CA in murine head and neck epithelium drives tumor invasion and metastasis through PDK1 and enhanced TGFβ signaling.. Oncogene.

[11] Marquard FE, Jücker M (2020). PI3K/AKT/mTOR signaling as a molecular target in head and neck cancer. Biochemical Pharmacology.

[12] Simpson DR, Mell LK, Cohen EE (2015). Targeting the PI3K/AKT/mTOR pathway in squamous cell carcinoma of the head and neck.. Oral Oncol.

[13] Johnson M, Sharma M, Henderson BR (2009). IQGAP1 regulation and roles in cancer.. Cell Signal.

[14] Brown MD, Sacks DB (2006). IQGAP1 in cellular signaling: bridging the GAP.. Trends Cell Biol.

[15] Smith JM, Hedman AC, Sacks DB (2015). IQGAPs choreograph cellular signaling from the membrane to the nucleus.. Trends Cell Biol.

[16] White CD, Brown MD, Sacks DB (2009). IQGAPs in cancer: a family of scaffold proteins underlying tumorigenesis.. FEBS Lett.

[17] Patel V, Hood BL, Molinolo AA, Lee NH, Conrads TP, Braisted JC, Krizman DB, Veenstra TD, Gutkind JS (2008). Proteomic analysis of laser-captured paraffin-embedded tissues: a molecular portrait of head and neck cancer progression.. Clin Cancer Res.

[18] Wu CC, Li H, Xiao Y, Yang LL, Chen L, Deng WW, Wu L, Zhang WF, Sun ZJ (2018). Over-expression of IQGAP1 indicates poor prognosis in head and neck squamous cell carcinoma.. J Mol Histol.

[19] Wei T, Choi S, Buehler D, Anderson RA, Lambert PF (2020). A PI3K/AKT Scaffolding Protein, IQ Motif-Containing GTPase Associating Protein 1 (IQGAP1), Promotes Head and Neck Carcinogenesis.. Clin Cancer Res.

[20] McNulty DE, Li Z, White CD, Sacks DB, Annan RS (2011). MAPK scaffold IQGAP1 binds the EGF receptor and modulates its activation.. J Biol Chem.

[21] Choi S, Hedman AC, Sayedyahossein S, Thapa N, Sacks DB, Anderson RA (2016). Agonist-stimulated phosphatidylinositol-3,4,5-trisphosphate generation by scaffolded phosphoinositide kinases.. Nat Cell Biol.

[22] Chen M, Choi S, Jung O, Wen T, Baum C, Thapa N, Lambert PF, Rapraeger AC, Anderson RA (2019). The Specificity of EGF-Stimulated IQGAP1 Scaffold Towards the PI3K-Akt Pathway is Defined by the IQ3 motif.. Sci Rep.

[23] Li S, Wang Q, Chakladar A, Bronson RT, Bernards A (2000). Gastric hyperplasia in mice lacking the putative Cdc42 effector IQGAP1.. Mol Cell Biol.

[24] Tang XH, Knudsen B, Bemis D, Tickoo S, Gudas LJ (2004). Oral cavity and esophageal carcinogenesis modeled in carcinogen-treated mice.. Clin Cancer Res.

[25] Jameson KL, Mazur PK, Zehnder AM, Zhang J, Zarnegar B, Sage J, Khavari PA (2013). IQGAP1 scaffold-kinase interaction blockade selectively targets RAS-MAP kinase-driven tumors.. Nat Med.

[26] Goto T, Sato A, Adachi S, Iemura S, Natsume T, Shibuya H (2013). IQGAP1 protein regulates nuclear localization of β-catenin via importin-β5 protein in Wnt signaling.. J Biol Chem.

[27] Nyman PE, Buehler D, Lambert PF (2018). Loss of Function of Canonical Notch Signaling Drives Head and Neck Carcinogenesis.. Clin Cancer Res.

[28] Beaty BT, Moon DH, Shen CJ, Amdur RJ, Weiss J, Grilley-Olson J, Patel S, Zanation A, Hackman TG, Thorp B, Blumberg JM, Patel SN, Weissler MC (2020). PIK3CA mutation in HPV-associated OPSCC patients receiving deintensified chemoradiation.. J Natl Cancer Inst.

